# A phase 1, open‐label study to evaluate the drug interaction between islatravir (MK‐8591) and the oral contraceptive levonorgestrel/ethinyl estradiol in healthy adult females

**DOI:** 10.1002/jia2.25858

**Published:** 2021-12-22

**Authors:** Wendy Ankrom, Deanne Jackson Rudd, Saijuan Zhang, Kerry L. Fillgrove, Kezia N. Gravesande, Randolph P. Matthews, Darin Brimhall, S. Aubrey Stoch, Marian N. Iwamoto

**Affiliations:** ^1^ Merck & Co., Inc. Merck Research Labs Kenilworth New Jersey USA; ^2^ PPD Development, Research Las Vegas Nevada USA

**Keywords:** drug–drug interaction, islatravir, antiretrovirals, hormonal contraceptive, levonorgestrel/ethinyl estradiol

## Abstract

**Introduction:**

Hormonal contraceptives are among the most effective forms of reversible contraception, but many other compounds, including some antiretrovirals, have clinically meaningful drug–drug interactions (DDIs) with hormonal contraceptives. Islatravir is a novel human immunodeficiency virus nucleoside reverse transcriptase translocation inhibitor currently in clinical development for treatment and prevention of HIV infection. A phase 1 clinical trial was conducted to evaluate the DDI of islatravir and the combination of oral contraceptive levonorgestrel (LNG)/ethinyl estradiol (EE).

**Methods:**

This was an open‐label, two‐period, fixed‐sequence, DDI clinical trial in healthy, postmenopausal or bilaterally oophorectomized females aged 18 through 65 years in the United States between October 2016 and January 2017. A single dose of LNG 0.15 mg/EE 0.03 mg was given followed by a 7‐day washout. Islatravir, 20 mg, was then dosed once weekly for 3 weeks; a single dose of LNG 0.15 mg/EE 0.03 mg was given concomitantly with the third dose of islatravir. Pharmacokinetic samples for plasma LNG and EE concentrations were collected pre‐dose and up to 120 hours post‐dose in each period. Safety and tolerability were assessed throughout the trial by clinical assessments, laboratory evaluations and examination of adverse events.

**Results and Discussion:**

Fourteen participants were enrolled. The pharmacokinetics of LNG and EE were not meaningfully altered by co‐administration with islatravir. For the comparison of (islatravir + LNG/EE)/(LNG/EE alone), the geometric mean ratios (GMRs) (90% confidence intervals [CIs]) for LNG AUC_0–inf_ and *C*_max_ were 1.13 (1.06, 1.20) and 0.965 (0.881, 1.06), respectively. For EE, the GMRs (90% CI) for AUC_0–inf_ and *C*_max_ were 1.05 (0.981, 1.11) and 1.02 (0.971, 1.08), respectively. Co‐administration of all three drugs was generally well tolerated.

**Conclusions:**

The results of this trial support the use of LNG/EE contraceptives in combination with islatravir without dose adjustment.

## 1 INTRODUCTION

In 2019, 38.0 million people were living with HIV globally, 52% of whom were women. Approximately 5500 young women aged 15–24 years become infected with HIV weekly worldwide. In sub‐Saharan Africa, among 15‐ to 24‐year olds, women are twice as likely to be living with HIV as men, and in adolescents aged 15–19, five of six new HIV infections occur in girls [1]. Pregnancy for women living with HIV, particularly unplanned, is associated with risks, including maternal mortality, potential perinatal transmission and teratogenicity associated with certain antiretroviral (ARV) regimens [2, 3, 4, 5]. Access to reliable and highly effective contraception for women living with HIV is critical [3]. Additionally, drug–drug interactions (DDIs) have been observed between some ARVs and hormonal contraceptives, limiting their use in women with reproductive potential [6, 7].

Islatravir (MK‐8591) is a nucleoside reverse transcriptase translocation inhibitor with potent antiviral activity against wild‐type and drug‐resistant HIV‐1 [8]. Islatravir is converted to the pharmacologically active triphosphate form (islatravir‐TP) and inhibits reverse transcriptase via multiple mechanisms [9]. Following a single oral administration, islatravir is rapidly absorbed with a median time to maximum concentration (*T*_max_) of 0.5 hours and a plasma half‐life (*t*_1/2_) of ∼50–60 hours [10]. Intracellular islatravir‐TP levels reach *T*_max_ between 6 and 24 hours and decline with a half‐life of ∼120–210 hours [10]. Following the administration of multiple weekly doses, there is minimal accumulation of islatravir in plasma [10]. Due to the unique pharmacokinetic profile, islatravir may be administered in a variety of dosing schedules from once daily to longer intervals. Unlike many other ARVs, islatravir is not an inhibitor or inducer of major cytochrome P450 (CYP) enzymes [11].

Hormonal contraceptives are the most widely used form of highly effective reversible contraception and typically contain a progestin with or without an estrogenic component. Levonorgestrel (LNG)/ethinyl estradiol (EE) is a popular fixed‐dose combination oral contraceptive. LNG does not undergo first‐pass metabolism, instead undergoing phase I metabolism likely mediated in part via CYP enzymes [12, 13]. First‐pass metabolism of EE occurs in the gut wall by sulfotransferase, and to a smaller extent, by CYP enzymes in the liver [12, 14]. Following oral administration, both LNG and EE are rapidly absorbed with a *T*_max_ of ∼1.5 hours. The terminal *t*_1/2_ of LNG and EE after single oral doses is approximately 34 and 18 hours, respectively [12].

The potential for clinically significant interactions between contraceptives and a number of ARVs can complicate the choice of both hormonal contraceptive and ARV regimen [15, 16]. Co‐administration with drugs that inhibit or induce the metabolizing enzymes of progestins and/or oestrogens may change plasma concentrations of these hormones, altering contraceptive efficacy and incidence of adverse events (AEs) [17, 18]. Islatravir does not inhibit or induce major metabolic enzymes or transporters [11]. Based on this, islatravir is not expected to alter the pharmacokinetics of hormonal contraceptives. For confirmation of these expected findings, a trial was conducted to evaluate the effect of islatravir on the pharmacokinetics of LNG and EE in healthy postmenopausal or bilaterally oophorectomized female participants to assess the suitability of combined use of islatravir with these hormonal contraceptives.

## 2 METHODS

### 2.1 Trial design

Protocol MK‐8591‐006 was an open‐label, two‐period, fixed‐sequence, DDI trial to assess the effects of multiple, once‐weekly oral doses of islatravir on the single‐dose pharmacokinetics of the combined oral contraceptive LNG/EE conducted in the United States between October 2016 and January 2017. The trial was conducted in accordance with principles of Good Clinical Practice and was approved by Novum Independent Institutional Review Board (Pittsburgh, PA).

### 2.2 Participant population

Eligible participants were healthy females 18 and 65 years, who were postmenopausal or bilaterally oophorectomized, had estradiol concentrations ≤35 pg/ml, had FSH levels ≥40 mIU/ml and had a body mass index (BMI) of 19–30 kg/m^2^. Levels of sex hormone binding globulin (SHBG) can fluctuate during the menstrual cycle, which affects the pharmacokinetics of hormonal contraceptives and can confound the interpretation of pharmacokinetic data. Therefore, this study enrolled healthy, postmenopausal or oophorectomized women to minimize any within participant variability in the pharmacokinetics of combination oral contraceptive pills, following once‐weekly administration of islatravir. Key exclusion criteria included pregnancy, lactation and clinically significant health conditions.

### 2.3 Treatments

Participants received a single dose of 0.15 mg LNG/0.03 mg EE [7] on day 1 (period 1) [7, 12]. After a minimum 7‐day washout interval, participants received 20 mg of islatravir once every 7 days for 3 weeks (day 1, day 8, and day 15). On day 15, 0.15 mg LNG/0.03 mg EE was co‐administered with the third dose of islatravir (period 2). The dose of 20 mg islatravir was selected as it is a dose within the projected therapeutic range for weekly administration [19, 20].

### 2.4 Assessments

Plasma for the measurement of LNG/EE concentration in period 1 was collected pre‐dose and 0.5, 1, 1.5, 2, 3, 4, 6, 8, 12, 24, 48, 72, 96, and 120 hours post‐dose. For period 2, plasma for the measurement of LNG/EE concentration was collected pre‐dose on day 15 and at the same timepoints as period 1 post‐dose. Safety and tolerability were assessed throughout the trial by clinical assessments, laboratory evaluations and examination of AEs.

### 2.5 Pharmacokinetic/pharmacodynamic analysis

The plasma concentrations of LNG and EE were determined by InVentiv Health Clinique, Inc. (Québec, Canada) (now Syneos Health) via a validated liquid chromatography‐tandem mass spectrometry assay. For LNG and EE, the calibration ranges were 10–10,000 pg/mL and 1–200 pg/mL, respectively. The pharmacokinetic parameter values were calculated using the software Statistical Analysis System (SAS^®^, Version 9.4). Plasma LNG and EE concentrations and actual sampling times were used to estimate LNG and EE pharmacokinetic parameters. *C*_max_ and *T*_max_ values were determined from the observed plasma concentration time data. AUC_0–last_ was calculated using the linear trapezoidal method for ascending concentrations and the log trapezoidal method for descending concentrations. AUC_0–∞_ for the analytes was calculated as AUC_0–last_+C_est_,_t_/Kel, where C_est,t_ is the concentration at the last blood sampling timepoint as predicted from the terminal‐phase linear regression. For each participant, Kel (λz) was estimated from the negative slope of the dataset with the best‐fit least‐squares linear regression analysis of the terminal ln‐linear concentration time data, and the apparent terminal *t*_1/2_ was calculated as ln(2)/λz. At least three data points in the terminal phase were used for Kel calculations.

### 2.6 Statistical analysis

Individual AUC_0–∞_ LNG and EE values were natural log‐transformed prior to analysis and evaluated separately using a linear mixed effects model with fixed effects term for treatment. An unstructured covariance matrix was used to allow for unequal treatment variances and to model the correlation between the two treatment measurements within each participant. Kenward and Roger's method was used to calculate the denominator degrees of freedom for the fixed effects. A two‐sided 90% confidence interval (CI) for the true mean difference (LNG/EE with islatravir minus LNG/EE alone) for each parameter on the log scale was computed from the above linear mixed effect model. The 90% CI on the log scale was exponentiated to obtain the 90% CI for the true geometric mean ratio (GMR) ([LNG/EE] with islatravir/[LNG/EE alone]) for each parameter. *C*_max_ was analysed in a similar fashion as AUC_0–∞_. Assuming 10 participants with available pharmacokinetic data, non‐negative correlation among the four test statistics and true GMRs of 0.95 for both parameters, there was at least 80% probability that both 90% CIs would satisfy their corresponding target intervals simultaneously.

## 3 RESULTS AND DISCUSSION

Fourteen women were enrolled and completed all dosing and pharmacokinetic evaluations. Safety evaluations were completed in all but one participant lost to follow‐up. Therefore, only 13 participants completed the trial. All 14 participants were included in the pharmacokinetic and safety analyses. Participants had a median age of 55.5 years (range 50–64) and a median BMI of 27.7 kg/m^2^ (range 21.3–29.6; Table 1).

**Table 1:** Summary of trial participant demographics

**Trial participants, *N***	14
**Sex, *n* (%)**	
Female	14 (100.0)
**Age (years)**	
Mean (SD)	54.7 (4.1)
Median (range)	55.5 (50–64)
**Weight (kg)**	
Mean (SD)	72.0 (9.2)
Median (range)	72.3 (56.0–91.0)
**BMI (kg/m^2^)**	
Mean (SD)	26.6 (2.5)
Median (range)	27.7 (21.3–29.6)
**Race** ^a^, ***n* (%)**	
Black or African American	8 (57.1)
White	3 (21.4)
White and Asian	1 (7.1)
Other	2 (14.3)
Ethnicity, *n* (%)	
Hispanic or Latino	5 (35.7)
Not Hispanic or Latino	9 (64.3)

^a^ Race was self‐reported by participants.

**Figure 1:**
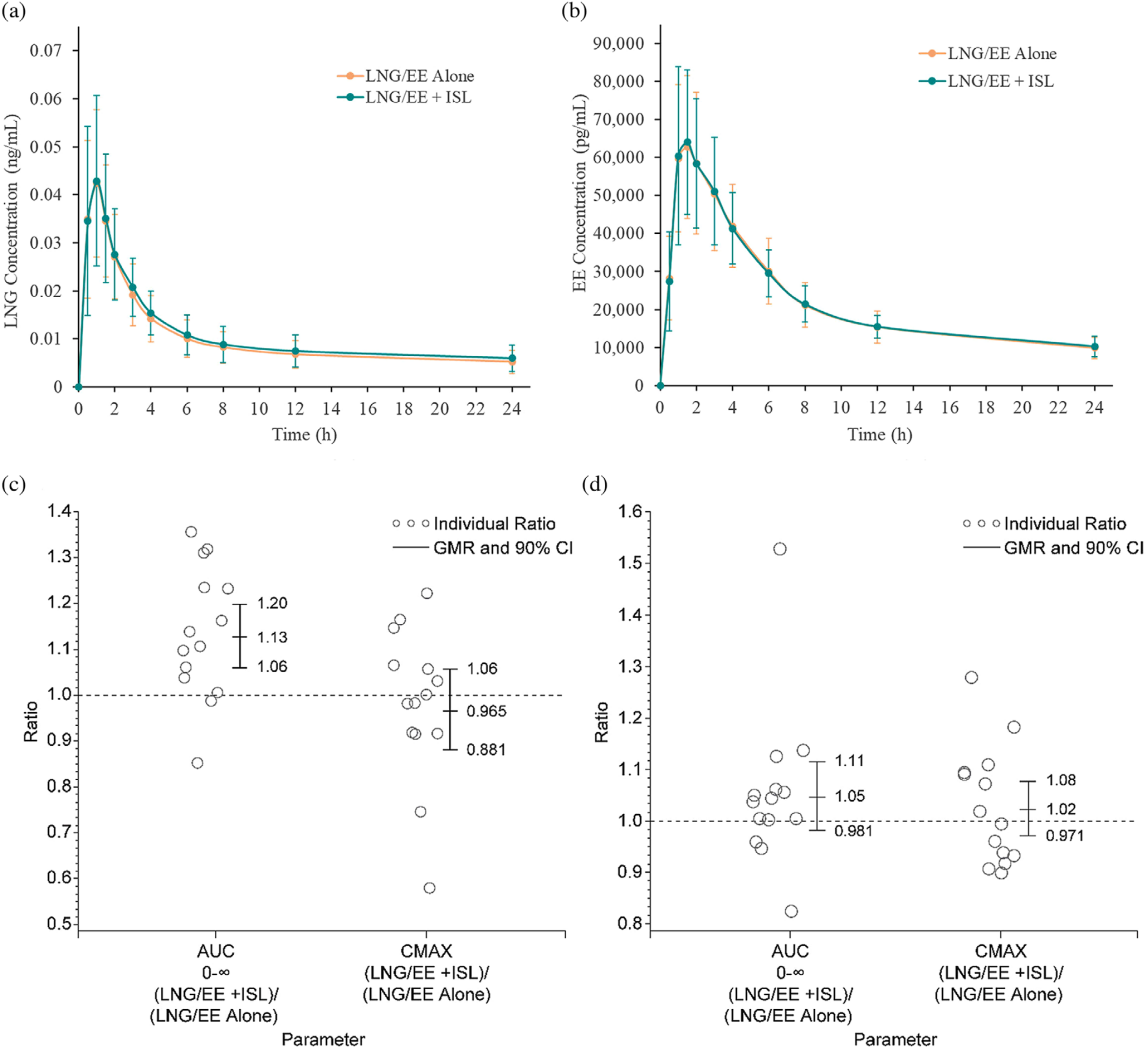
(a) Mean linear (± SD) plasma concentration of LNG versus time following administration of a single dose of LNG/EE (0.15/0.03 mg) with or without co‐administration of multiple weekly doses of 20 mg ISL. (b) Mean linear (± SD) plasma concentration of EE versus time following administration of a single dose of LNG/EE (0.15/0.03 mg) with or without co‐administration of multiple weekly doses of 20 mg ISL. (c) Individual and geometric mean ratios and 90% CI of LNG AUC_0–∞_ and *C*_max_. (d) Individual and geometric mean ratios and 90% CI of EE AUC_0–∞_ and *C*_max_. Abbreviations: EE, ethinyl estradiol; GMR, geometric mean ratio; ISL, islatravir; LNG, levonorgestrel.

For LNG, the GMR for AUC_0–inf_ was 13% higher with LNG/EE co‐administered with islatravir compared to LNG/EE alone. LNG *C*_max_, apparent terminal *t*_1/2_ and *T*_max_ were similar for both treatments (Figure 1 and Table 2). For EE, AUC_0–inf_, *C*_max_, apparent terminal *t*_1/2_ and *T*_max_ were similar for both treatments (Figure 1 and Table 2).

**Table 2:** Summary of plasma pharmacokinetics for LNG and EE following a single dose of LNG/EE with or without co‐administration of ISL

	LNG/EE alone^a^		LNG/EE + ISL^b^				
Pharmacokinetic parameter	*N* = 14		*N* = 14		(LNG/EE + ISL)/(LNG/EE alone)		
LNG	GM^c^	95% CI^c^, ^e^	GM^c^	95% CI^c^, ^e^	GMR	90% CI	Within participant CV (%)^d^
AUC 0–∞ (ng/ml·h)	44.4	(34.4, 56.9)	50.0	(39.4, 63.4)	1.13	(1.06, 1.20)	9.20%
*C*_max_ (ng/ml)	3.56	(2.83, 4.44)	3.44	(2.64, 4.44)	0.965	(0.881, 1.06)	13.60%
*T*_max_ (h)^c^	1	(0.50, 1.50)	1	(0.50, 1.50)			
*T*_1/2_ (h)^e^	34.48	26.2	38.22	21.1			

EE	GM^c^	95% CI^c^, ^e^	GM^c^	95% CI^c^, ^e^	GMR	90% CI	Within participant CV (%)^d^
AUC 0–∞ (pg·h/ml)	788	(651, 953)	824	(713, 952)	1.05	(0.981, 1.11)	9.50%
*C*_max_ (pg/ml)	61.7	(51.3, 73.8)	62.8	(52.8, 75.0)	1.02	(0.971, 1.08)	7.60%
*T*_max_ (h)^c^	1.5	(1.00, 2.00)	1.5	(1.00, 2.00)			
*T*_1/2_ (h)^e^	20.39	15.6	20.71	12.6			

*Note*: All estimates for AUC_0–∞_ and *C*_max_ are based upon the linear mixed‐effects model. Statistics for other parameters are calculated directly.

^a^ Single dose of LNG/EE tablet (0.15/0.03 mg).

^b^ ISL 20 mg (2×10 mg capsules) once weekly for 3 weeks with a single dose of LNG/EE tablet (0.15/0.03 mg) on day 15.

^c^ Median (Min, Max) reported for *T*_max_.

^d^ Within‐participant CV(%) estimated based on the elements of the variance‐covariance matrix: CV(%) = 100*sqrt[(s^2^_A_ + s^2^_B_ –2*s_AB_)/2].

^e^ Geometric CV(%) is reported for *t*_1/2_.

Administration of LNG/EE alone and with islatravir was generally well tolerated. There were no deaths or serious AEs reported. Seventeen treatment‐emergent AEs were reported by 7 of 14 participants; three occurred after administration of LNG/EE alone, eight occurred after administration of islatravir alone and six occurred after co‐administration of LNG/EE and islatravir. All reported AEs were mild in intensity and resolved by the end of trial. The most common AEs (occurring in two or more participants) were abnormal urinalysis (*n* = 2, 14.3%) and pruritus (*n* = 2, 14.3%); none of which were considered by the investigator to be drug related.

The results of this trial demonstrate that co‐administration of multiple doses of islatravir with a single dose of LNG/EE does not have a clinically meaningful effect on the pharmacokinetics of LNG or EE. The 90% CI of LNG and EE AUC_0–inf_ and *C*_max_ fall within bioequivalence bounds [0.8, 1.25]. A minor increase in LNG AUC_0–inf_ of 13% was seen but was not considered clinically relevant. The mechanism behind this minor increase is unknown. Co‐administration of islatravir with LNG/EE was generally well tolerated. Overall, these results suggest that islatravir would be suitable for co‐administration with hormonal contraceptives containing LNG and/or EE. The long *t*_1/2_ of islatravir‐TP supports varying dosing schedules, spanning from daily administration to more extended intervals.

This trial was conducted with a 20 mg islatravir dose, which is expected to achieve therapeutic weekly concentrations as well as relevant concentrations for DDI assessments. The effect of islatravir on LNG/EE was tested after multiple once weekly doses to rule out any time‐dependent inhibition or induction, although none was anticipated based on the in vitro DDI assessment for islatravir. Two doses of islatravir were administered prior to the co‐administration of islatravir with LNG/EE on day 15 to ensure that any potential inductive effect of islatravir had reached steady state prior to co‐administration with LNG/EE and subsequent pharmacokinetic analysis.

Postmenopausal and bilaterally oophorectomized women were enrolled in this trial, as this group would not have the cyclical fluctuations in SHBG observed in ovulatory women [21]. LNG binds to SHBG, thus changes in SHBG levels would alter clearance and affect oral contraceptive DDI assessments in ovulatory women [12]. A potential DDI leading to changes in LNG/EE pharmacokinetics due to an effect on metabolic enzymes would be the same in anovulatory and ovulatory women. To reduce variability in oral contraceptive pharmacokinetics related to fluctuations in SHBG during the menstrual cycle, oral contraceptive DDI studies are frequently conducted in anovulatory women, with the results being applied to ovulatory women (ie women of childbearing potential) [22, 23, 24, 25].

Oral hormonal contraceptives are generally taken daily, whereas this study only assessed single‐dose administration. The magnitude of effect of ISL on LNG and EE PK would not be expected to vary with multiple doses of LNG/EE based on the pharmacokinetic profiles of LNG and EE; therefore, the results of this study can be extrapolated to multiple‐dose administration of hormonal contraceptives [25]. Atogepant and doravirine both used a single‐dose approach [22, 24]. Another limitation of this trial is the fixed sequence design. Due to the long half‐life of islatravir, a cross over design would have been operationally complex. The period effect should not meaningfully impact the results.

## 4 CONCLUSIONS

These results together with information on lack of effect of islatravir on major CYP enzymes and transporters suggest that islatravir is unlikely to meaningfully affect the pharmacokinetics of hormonal contraceptives [11]. This finding supports the use of hormonal contraceptives in combination with islatravir without dose adjustment. Although this trial evaluated the effect of islatravir on an orally administered hormonal contraceptive, the lack of a pharmacokinetic effect supports the application of these findings to other forms of LNG (e.g. implants).

## COMPETING INTERESTS

WA, DJR, SZ, KF, KG, RPM, SAS and MI are employees or former employees of Merck Sharp & Dohme Corp., a subsidiary of Merck & Co., Inc., Kenilworth, NJ, USA, who may own stock and/or hold stock options in the Company. DB has no conflicts to report.

## AUTHORS’ CONTRIBUTIONS

WA contributed to the conception, design/planning of the study, analysis of data, acquisition of data, interpretation of results, drafting the manuscript and critically reviewing/revising the manuscript for intellectual content. MI contributed to the interpretation of results and critically reviewing/revising the manuscript for intellectual content. AS contributed to the acquisition of the data, interpretation of results and critically reviewing/revising the manuscript for intellectual content. SZ contributed to the conception, design/planning of the study, analysis of data, interpretations of results and critically reviewing/revising the manuscript for intellectual content. JD contributed to the conception, design/planning of the study, acquisition of the data, interpretation of results and critically reviewing/revising the manuscript for intellectual content. KF contributed to the conception, design or planning of the study and critically reviewing/revising the manuscript for intellectual content.

MR contributed to the analysis of the data, interpretation of results and critically reviewing/revising the manuscript for intellectual content. DJ contributed to the conception, design or planning of the study, interpretation of the results and critically reviewing/revising the manuscript for intellectual content. DB contributed to the conception, design or planning of the study, acquisition of the data, interpretation of the results and critically reviewing/revising the manuscript for intellectual content. KG contributed to the conception, design or planning of the study and critically reviewing/revising the manuscript for intellectual content.

## FUNDING

Merck Sharp & Dohme Corp., a subsidiary of Merck & Co., Inc., Kenilworth, NJ, USA, provided financial support for the trial.

## DATA SHARING STATEMENT

Merck Sharp & Dohme Corp., a subsidiary of Merck & Co., Inc., Kenilworth, NJ, USA (MSD), is committed to providing qualified scientific researchers access to anonymized patient‐level data and clinical study reports from the company's clinical trials for the purpose of conducting legitimate scientific research. The company is also obligated to protect the rights and privacy of trial participants and, as such, has a procedure in place for evaluating and fulfilling requests for sharing company clinical trial data with qualified external scientific researchers. The process includes submission of data requests to the MSD data sharing website available at: http://engagezone.msd.com/ds_documentation.php. Data will be made available for request after product approval in the United States and EU or after product development is discontinued. There are circumstances that may prevent MSD from sharing the requested data.
